# A rare case of idiopathic giant bullous emphysema or vanishing lung syndrome

**DOI:** 10.11604/pamj.2024.48.121.43872

**Published:** 2024-07-19

**Authors:** Aishwarya Kishor Kedar, Vivek alone

**Affiliations:** 1 Department of Respiratory Medicine, Datta Meghe Institute of Higher Education and Research, Wardha, Maharashtra, India

**Keywords:** Giant apical bulla, emphysematous changes, idiopathic giant bullous emphysema

## Images in medicine

A thirty-eight-year-old female presented with the complaints of fever, cough with expectoration and breathlessness on exertion since 2 months. There was no history of pulmonary tuberculosis, other infections or any comorbid condition. She had a history of tobacco chewing since 20 years. On examination she was conscious and oriented to time, place and person, pulse rate was 110/minute, respiratory rate was 26/minute, saturation was 89% on room air, blood pressure was 120/80 mmHg, febrile (100.4° Fahrenheit). Her systemic examination revealed bilaterally decreased breath sounds with hyperresonant note on right side of chest. Her arterial blood gas analysis was suggestive of type I respiratory failure. Sputum examination was negative for acid-fast bacilli. Chest radiography was done which revealed a large air space with air fluid level in right upper zone. High resolution computed tomography of the lungs was further carried out for the patient which showed avascular, giant apical bulla with an air fluid level, compressing surrounding lung parenchyma on right side. There were panacinar and paraseptal emphysematous changes in bilateral lung fields. Her laboratory investigations revealed a total leukocyte count of 18000/mm^3^. She tested negative for hepatitis B, hepatitis C and human immunodeficiency virus. Serologic testing for connective tissue disease was negative. Alfa-1 antitrypsin concentration was 1.0 g/L which was also within normal range. A diagnosis of vanishing lung syndrome was made. Vanishing lung syndrome is a rare condition causing formation of giant emphysematous bullae in upper lobes and occupies at least one-third of one or both hemithoraces causing compression of surrounding lung parenchyma also known as idiopathic giant bullous emphysema. Patient was treated with antibiotics and supportive treatment and was referred for Video-Assisted Thoracoscopic Surgery with bullectomy.

**Figure 1:**
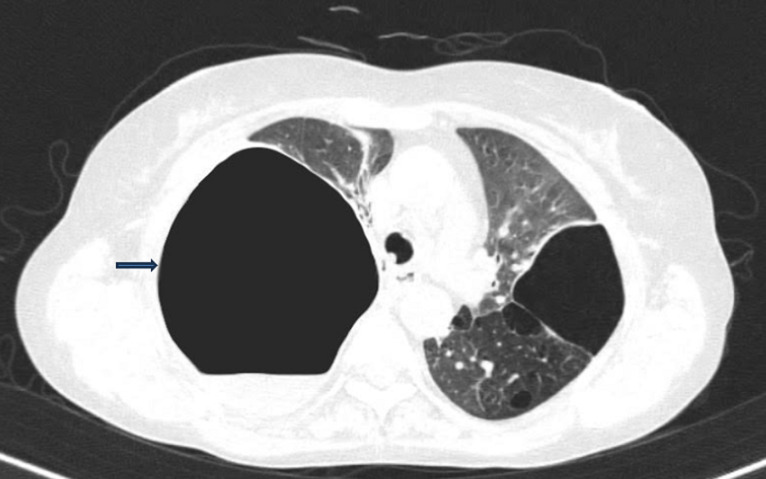
an axial section of computed tomography of lungs showing a giant apical bulla on the right side with air-fluid level inside and compressing surrounding lung parenchyma

